# Harmonisation of the HLA tests for the diagnosis of coeliac disease: experiences from the Czech external proficiency testing program

**DOI:** 10.3389/fgene.2024.1441769

**Published:** 2024-09-09

**Authors:** Milena Vrana, Jana Tajtlova, Frantisek Mrazek

**Affiliations:** ^1^ Department of HLA, Institute of Hematology and Blood Transfusion, Prague, Czechia; ^2^ Department of Medical Genetics, Thomayer University Hospital, Prague, Czechia; ^3^ HLA laboratory, Department of Immunology, University Hospital, Olomouc, Czechia

**Keywords:** coeliac disease, HLA, external proficiency testing, disease association, genetic susceptibility

## Abstract

Coeliac disease (CD) is an autoimmune disorder caused by the ingestion of gluten-containing grains. One of the prerequisites for the development of the disease is the presence of specific combinations of HLA alleles at the DQA1 and DQB1 loci. The HLA test is a supportive diagnostic test. In the Czech Republic, approximately 3,500 HLA tests for CD diagnosis are performed annually in almost three dozen laboratories. The HLA Department of the Institute of Haematology and Blood Transfusion in Prague has been offering the EPT “Detection of HLA Alleles Associated with Diseases” for more than 10 years. The results are evaluated in terms of the correct determination of predisposing alleles/allelic groups and clinical interpretation. Every year, we notice some problems with the detection of CD-associated alleles and the interpretation of results. Annual workshops are part of this EPT, and they also include recommendations for the interpretation of results. This interpretation is evolving based on the current knowledge in the field. The current recommendation for interpretation was adopted in 2023, dividing HLA-DQA1/DQB1 genotypes into three categories: 1) detected HLA genotype is associated with predisposition to coeliac disease; 2) coeliac disease could not be excluded based on the detected HLA genotype; 3) coeliac disease could be excluded with high probability based on the detected HLA genotype. The quality of examination is increasing but still needs improvement. Correct results and accurate interpretation can inform clinicians’ decisions about the diagnosis of coeliac disease in appropriate patients.

## Introduction

Coeliac disease (CD) is an autoimmune disorder triggered by the ingestion of gluten, primarily affecting the small intestine (Catassi et al., 2022). The average prevalence of CD is approximately 1% in the populations of developed countries. The clinical manifestations of CD are highly variable and may combine a wide range of gastrointestinal and extraintestinal symptoms, including those of malabsorption. Diagnosis is based on clinical suspicion, serological parameters (positivity for anti-transglutaminase 2 and anti-endomysial antibodies), and, if necessary, a biopsy of the small intestine (Husby et al., 2020). Despite current efforts to develop an effective tolerance-inducing therapy for CD (Sollid, 2024), a lifelong gluten-free diet remains the essential tool for disease control and, importantly, preventing CD complications such as growth retardation, anaemia, osteoporosis, and intestinal lymphoma.

The pathogenesis of CD is based on the interplay between the genetically predisposed individual and (mostly unknown) environmental factors (Levescot et al., 2022). Accordingly, CD is a complex disease with a strong genetic component in which specific disease-associated HLA-DQ variants present deamidated gluten peptides to the specific CD4^+^ T lymphocytes that finally trigger the disease (Sollid, 2024). Historically, the first report of an association between the HLA system and CD was published as early as 1973 (Evans, 1973), although this study only identified the HLA marker (HLA-B8) in linkage disequilibrium and did not identify a causal HLA variant for CD (Mrazek, 2023). In the following decades, studies using new methods of HLA testing based on DNA analysis made it possible to precisely specify the HLA variants necessary for the development of CD, namely HLA-DQ2 (heterodimers DQ2.5 and DQ2.2) and DQ8. Furthermore, it has been clearly shown that not only the presence of these CD-predisposing HLA variants but also their genotype status (gene-dose effect) and/or mutual homologous combinations contribute to the overall risk of the disease (Megiorni and Pizzuti, 2012; Erlichster et al., 2020).

Identification of the associated HLA-DQ variants has been widely incorporated into the diagnosis of CD, although it is not currently mandatory for diagnosis (Husby et al., 2020). Current approaches to certified laboratory HLA tests in CD aim to detect the HLA allelic combinations that predispose people to the disease. Importantly, these tests are characterised by a high negative predictive value—an absence of the predisposing HLA variants in the subject has a high probability of excluding CD.

The aim of this article is to briefly summarise the long-term experience with the external proficiency testing program (EPT) “Detection of HLA Alleles Associated with Diseases” organised by the HLA department of the Institute of Haematology and Blood Transfusion in Prague. Therefore, presented and discussed here are the characteristics of the EPT program, the development of national guidelines for the interpretation of the HLA testing in CD in the context of international recommendations, the main problems in the HLA genotyping and interpretation of the results by the EPT participants, and the current directions in genetic testing for CD.

## External proficiency testing program “Detection of HLA Alleles Associated with Diseases”

Approximately 3,500–4,000 HLA tests for CD diagnosis are performed annually in almost three dozen laboratories in the Czech Republic; details of the system of HLA tests for CD have been published elsewhere (Vraná et al., 2017). CE-IVD diagnostic kits for typing HLA genotypes predisposing for CD are used by the laboratories in the majority of reported results.

The HLA department of the Institute of Haematology and Blood Transfusion has organised the EPT “Detection of HLA Alleles Associated with Diseases” since 2010. The EPT program is open to all willing laboratories and diagnostic kit providers/vendors. Any DNA-based method can be used by the EPT participants. Tests for the detection of HLA alleles associated with CD have always been part of this EPT program. Since 2012, we have organised and evaluated this EPT on a regular basis. The results have been evaluated in terms of the correct determination of predisposing HLA alleles/allele groups and their clinical interpretation. The correct detection of HLA-DQA1*02, *05 and *03; DQB1*02, and *03:02 is required for the EPT. The organising laboratory has provided reference DQA1 and DQB1 genotypes based on SBT/NGS high-resolution typing. Clinical interpretation is scored according to the current recommendations (see “Recommendations” section for the interpretation of HLA testing in the diagnosis of coeliac disease). Scoring details are specified in Supplementary Image S1.

A total of 13 Czech laboratories participated in the EPT in 2012, while 34 laboratories from six countries participated in 2023. Since 2013, the EPT has been divided into two rounds of five samples each; labs may choose to participate in one or both rounds. All participants are listed in Supplementary Data Sheet S1. Laboratory techniques used for the detection of associated HLA variants have changed over the years. Polymerase chain reaction with sequence-specific primers (PCR-SSP) has been the most commonly used method throughout the whole period. Details of the techniques used by participants in the most recent EPT year evaluated (2023) and associated genotyping errors are described below. All tests were performed using CE-IVD commercial kits from different manufacturers.

In 2023, various polymerase chain reaction kits with sequence-specific primers (PCR-SSP) intended for CD detection were used by 19 laboratories, one of which missed DQB1*03:02 in one sample. CD-specific real-time polymerase chain reaction (RT-PCR) kits were used by 13 laboratories, one of which incorrectly reported the presence of DQB1*03:02 instead of DQB1*03:03. Sequence-specific oligoprobe (SSO) CD-specific kits with various configurations, including microarray technology, were used by 12 laboratories, two of which incorrectly reported the presence of DQB1*03:02 instead of DQB1*03:03 (both errors were associated with a strip assay). Several laboratories reported using more than one method. Accordingly, the laboratories participating in both rounds were counted twice for the evaluation, as some of them used different methods in the first and second rounds.

**FIGURE 1:**
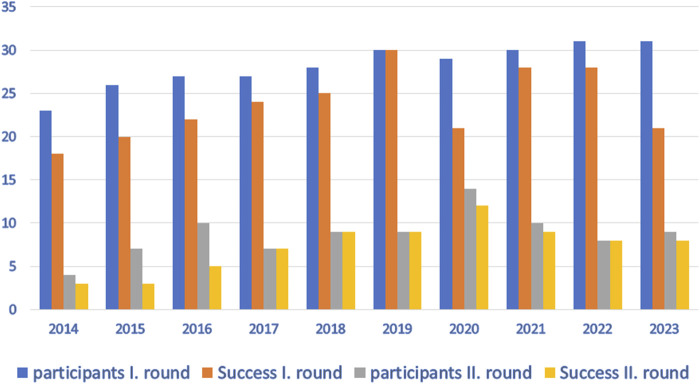
Participation and success in EPT in the last 10 years.

Every year (except 2019), we have noticed some problems with the detection of CD-associated alleles and the interpretation of the results. The number of participants and successful participation in the EPT program over the last 10 years is shown in Figure 1. The critical problem that repeatedly appeared in allele detection is the correct determination of the predisposing DQB1*03:02 variant. This allele is often incorrectly reported in individuals carrying the non-predisposing DQB1*03:03 allele. This error leads to the incorrect clinical interpretation of the results. Importantly, many other errors in HLA typing occur despite the use of CE-IVD diagnostic kits by the participating laboratories.

In our experience, the distinct year-to-year variability in EPT outcomes, as measured by the proportion of discrepancies, is related to the selection of samples for a particular EPT run. For example, the samples with uncommon linkage between the variants at HLA-Class II loci (rare haplotypes) tend to be reported with an error more frequently. The proportion of participating laboratories with discrepancies in EPT according to the particular DNA techniques used for testing EPT samples is presented in Table 1. Although we observed more errors among the limited number of laboratories using RT-PCR with one specific kit in the early years of the program, no further evidence of a significant relationship between the success rate in EPT and the techniques used was found.

**TABLE 1:** Overview of methods used by EPT participants and error rate in distinct years.

year→	2014		2015		2016		2017		2018	
method↓	labs (n)	errors (%)	labs (n)	errors (%)	labs (n)	errors (%)	labs (n)	errors (%)	labs (n)	errors (%)
PCR-SSP	13	7.7%	19	15.8%	17	5.9%	18	5.6%	23	8.7%
RT-PCR	1	100%	4	25.0%	5	100%	2	100%	0	N/A
SSO	9	22.2%	11	9.1%	15	33.3%	10	0.0%	17	0.0%
Sanger seq	0	N/A	0	N/A	0	N/A	1	0.0%	1	0.0%

year→	2019		2020		2021		2022		2023	
method↓	labs (n)	errors (%)	labs (n)	errors (%)	labs (n)	errors (%)	labs (n)	errors (%)	labs (n)	errors (%)
PCR-SSP	17	0.0%	20	15.0%	23	4.3%	19	10.5%	19	5.3%
RT-PCR	2	0.0%	5	0.0%	6	0.0%	11	18.2%	13	7.7%
SSO	11	0.0%	16	18.8%	10	0.0%	10	0.0%	12	16.7%
Sanger seq	0	N/A	0	N/A	0	N/A	0	N/A	0	N/A

year→	all years	
method↓	labs (n)	errors (%)
PCR-SSP	188	8.0%
RT-PCR	49	26.5%
SSO	121	10.7%
Sanger seq	2	0.0%

Error rate: 0% , 0.1%-9.9% , 10-19.9% , 20%-99.9% , 100% .

## Recommendations for the interpretation of HLA tests for the diagnosis of coeliac disease

Open annual workshops that are attended by the laboratories (EPT program participants), clinicians, and the representatives of diagnostic kit manufacturers are considered an integral part of the EPT program and also address recommendations for the interpretation of results. The interpretation of HLA results for CD is evolving based on current knowledge in the field.

The main goal of these recommendations is to pinpoint important facts regarding the predictive value of the HLA tests in CD.

• The frequency of HLA haplotypes with confirmed risk of CD in the Czech population is as follows: …DQA1*05/DQB1*02 aprox. 9.2%; DQA1*02/DQB1*02 aprox. 8.3%; DQA1*03/DQB1*03:02 aprox. 6.9%…. (Zajacova et al., 2016), altogether reaching 24.4%. Accordingly, we estimate that the proportion of individuals carrying at least one of these predisposing HLA-DQ haplotypes in the general Czech population is approximately 43%.
• The prevalence of CD in Western countries is approximately 1% (Catassi et al., 2022; Al-Toma et al., 2019).
• The presence of predisposing HLA alleles/haplotypes cannot be used to confirm CD, as HLA testing as such has low specificity and positive predictive value (Elwenspoek et al., 2021).
• On the other hand, the number of patients with confirmed CD who are not carriers of these alleles/haplotypes is very small, and therefore the examination has a high negative predictive value (Erlichster et al., 2020).

The first of our recommendations was established in 2015 and mainly focuses on consensus on the correct and complete issuance of diagnostic predisposition test results (Vraná et al., 2017). DQ2.5 and DQ8 were determined as the variants associated with the predisposition to CD in accordance with the European Society for Paediatric Gastroenterology, Hepatology, and Nutrition Guidelines for the Diagnosis of Coeliac Disease (Husby et al., 2012). Our recommendation was updated in 2020. Based on HLA analysis of the Czech cohort presented at the 2016 ESPGHAN Congress, DQ2.2 was established as “…associated with a rare risk of CD, the diagnosis could not be excluded”. In the update in 2022, the consensus was that DQ2.5, DQ2.2, and DQ8 variants are predisposing to CD, and the strength of the association conferred by different variants (e.g., DQ2.5 *versus* DQ2.2) is not considered for the interpretation of the tests. Recently, the genotype containing HLA-DQA1*05 without DQB1*02 or DQ8 was detected in two unrelated Czech individuals with the unequivocally confirmed diagnosis of coeliac disease. Both cases were presented in detail at the 2023 EPT annual workshop. This finding, together with the previous reports describing rare CD cases that were negative for established predisposing HLA variants, namely DQ2.5, DQ8, and DQ2.2, but positive only for DQA1*05 led to much discussion about the relevance of such genotypes for disease prediction. To reflect these observations, in 2023, we finally modified the recommendation on the interpretation of HLA tests for CD predisposition to its current version as follows:

• Identification of HLA-DQ2.5 (DQB1*02 + DQA1*05), DQ2.2 (DQB1*02 + DQA1*02 without DQA1*05), and/or DQ8 (DQB1*03:02 + DQA1*03) should be interpreted as “Detected HLA genotype is associated with the risk of CD. This result cannot be separately interpreted as a confirmation of CD due to low specificity.”
• HLA-DQA1*05 solely without any of the other predisposing alleles mentioned in the previous paragraph should be interpreted as “Detected HLA genotype is associated with the occurrence of coeliac disease in rare cases. The diagnosis of CD cannot be excluded.”
• Any combined HLA-DQB1, -DQA1 genotype other than those mentioned above should be interpreted as “Detected HLA genotype is not associated with the risk of CD. The result excludes this diagnosis with high probability.”

The recommendation is also concerned with the complete issuance of the results, including all information important for clinical use in a form that is easily understandable to the ordering clinician. A complete report should include the following key information.

• Detected HLA-DQA1 and DQB1 alleles/allele groups predisposing to CD.
• Information on the presence or absence of serological equivalents DQ2.5, DQ2.2, and DQ8.
• Clinical interpretation of the detected result as described above.

## Discussion

The detection of predisposing HLA variants has become an important part of the laboratory diagnosis of CD. Compared to the majority of other clinically relevant HLA disease associations, susceptibility to CD is conferred by the presence of specific combinations of alleles at two HLA loci (HLA-DQB1 and -DQA1) and therefore requires an appropriate approach for HLA genotyping and relevant interpretation for clinicians (Tye-Din et al., 2015).

The existence of various approaches for the genotyping, interpretation, and reporting of the results of HLA variants associated with CD that did not historically provide sufficient support for CD diagnostics highlights the need for the harmonisation of HLA diagnostics in CD. External proficiency testing (EPT) programs thus represent a crucial tool in the process of standardisation of HLA disease association testing in CD. Based on their ability to monitor methods, reagents (usually commercial kits), ways of reporting results, and errors for participating laboratories, EPT programs provide a valuable source of data and feedback for all entities involved in CD diagnostics and contribute to further overall improvement of HLA laboratory diagnostics in CD. For example, experience from our EPT program has already contributed to the modification of commercial kits (both in terms of technology and interpretation) to improve their ability to correctly assess genetic susceptibility to CD and to improve parameters of the reports from participating laboratories, such as the use of current HLA nomenclature and unambiguous/harmonised clinical interpretation that can be widely shared among clinicians.

Not surprisingly, due to the abovementioned complex nature of HLA’s association with CD and its interpretation, this part of our EPT program is consistently characterised by the highest proportion of errors in the results compared with other HLA tests offered by our program (e.g., HLA-B*27, -B*57:01, and -DQB1*06:02); similar experiences with poorer performance in HLA testing for CD are reported from other EPT programs that provide tests for HLA disease associations. An important example of a frequent and rather systematic error with a potentially detrimental effect on CD diagnosis is the false positive reporting of the predisposing HLA-DQB1*03:02 allele in subjects carrying only DQB1*03:03, which is not associated with CD.

It is important to note that various genetic laboratories (not just “HLA-oriented”) perform HLA-based CD genetic susceptibility testing, usually using commercial kits that focus on detecting combinations of CD-predisposing HLA alleles but do not provide a complete HLA genotype. Furthermore, a significant proportion of these laboratories are not involved in HLA testing for other clinical purposes, such as histocompatibility testing for transplantations, which requires the complete genotyping of specific HLA loci. In this context, the question may arise as to whether newer HLA typing techniques that provide a complete HLA genotype at a high-resolution level (e.g., next-generation sequencing) should be preferred to the CD-specific HLA kits (Devriese et al., 2024). Full HLA genotyping is a relevant and promising alternative for disease association testing and offers several clear advantages, such as the possibility of second-level reference typing in “problematic” samples or the assessment of gene dose effect. On the other hand, when properly and accurately used, commercial CD-specific kits can provide sufficient results to assess CD predisposition in the majority of samples in accordance with the above recommendations. However, as our results show, critical examination of these kits is necessary, and further development is needed. In the case of inconclusive results, alternatives should be sought, such as more precise methods for complete genotyping of the relevant loci as mentioned above. EPT, kit improvement, harmonisation of interpretation results, education, and the use of advanced technologies for sample processing and result evaluation (special applications, artificial intelligence, etc.) can contribute to error prevention and a more accurate and correct interpretation.

At present, there is wide agreement on the recommendations regarding the HLA variants predisposing to CD: heterodimers DQ2.5 (DQB1*02, DQA1*05), DQ2.2 (DQB1*02, DQA1*02) and DQ8 (DQB1*03:02, DQA1*03) (Tye-Din et al., 2015; Pritchard et al., 2024). Currently, the main purpose of HLA investigations in CD is to exclude the diagnosis in patients who do not carry the abovementioned relevant HLA-DQ heterodimers regardless of their gene dosage (heterozygosity/homozygosity) or their combinations. In other words, HLA tests in CD are used for their strong negative predictive value—that is, the ability to exclude the disease.

On the other hand, there is no complete consensus on the interpretation of the HLA-DQ genotypes containing only the HLA-DQA1*05 variant without any other predisposing alleles. HLA-DQA1*05, which encodes half of the DQ2.5 heterodimer, is a very common allelic group in Caucasoid populations. Importantly, for instance, the Australian position paper on the appropriate clinical use of HLA typing for CD (Tye-Din et al., 2015) recommends interpreting the genotype containing only HLA-DQA1*05 as being associated with a very low genetic susceptibility to CD, with the consideration of further clinical work-up for CD. Because we are also aware of rare cases of CD associated with genotypes containing HLA-DQA1*05 in the Czech population, our latest national recommendation for HLA testing in CD from 2023 complies with the above-mentioned Australian paper. In contrast, the very recent UK NEQAS and BSHI guidelines on HLA genotyping to support the diagnosis of CD recommend that individuals should be excluded from a diagnosis of CD if they carry only DQA1*05 or any other HLA-DQ alleles other than those encoding the heterodimers DQ2.5, DQ2.2, and DQ8. However, the question of how to deal with the interpretation of HLA-DQA1*05 solely positive individuals is still a matter of intensive debate (Maimaris et al., 2024).

## Conclusion

We have presented here our experience in HLA testing for CD from the external proficiency testing program (EPT) “Detection of HLA Alleles Associated with Diseases” organised by the HLA department of the Institute of Haematology and Blood Transfusion in Prague. The main goal of the program and associated annual workshops is to contribute to the standardisation, harmonisation, and improvement of the overall quality of the HLA investigations for the diagnosis of CD.
