# Supplementary figures and images for: Harmonisation of the HLA tests for the diagnosis of coeliac disease: experiences from the Czech external proficiency testing program
Source: Front Genet. 2024 Sep 9;15:1441769. doi: 10.3389/fgene.2024.1441769 (PMC11416978; doi:10.3389/fgene.2024.1441769)

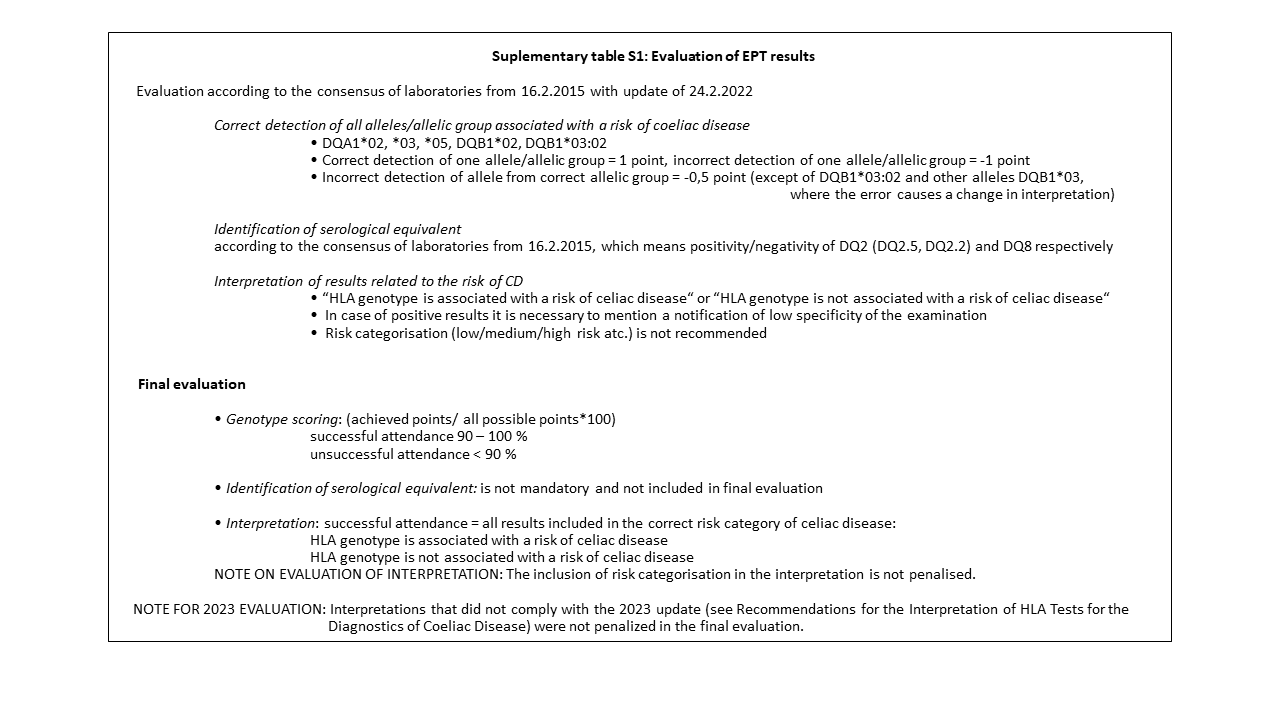

Supplement: Supplementary file 1 [file Image1.tif]
