# Supplementary material for: Harmonisation of the HLA tests for the diagnosis of coeliac disease: experiences from the Czech external proficiency testing program
Source: Front Genet. 2024 Sep 9;15:1441769. doi: 10.3389/fgene.2024.1441769 (PMC11416978; doi:10.3389/fgene.2024.1441769)
Supplement: Supplementary file 2 [file DataSheet1.docx]

| **Supplement S2:**  List of participants of external proficiency testing “[Detection of HLA Alleles Associated with Diseases](http://www.uhkt.cz/laboratories/external-proficiency-testing/detection-of-hla-alleles-associated-with-diseases)” organized by Institute of Hematology and Blood Transfusion | | |
| --- | --- | --- |
| **Laboratory** | **City** | **Country** |
| IMALAB s.r.o., Laboratoř molekulární biologie | Zlín | Czech Republic |
| Krajská nemocnice T. Bati, a.s., Laboratoř molekulární diagnostiky a cytogenetiky, | Zlín | Czech Republic |
| CGB laboratoř, a.s. | Ostrava | Czech Republic |
| Krevní centrum FN Ostrava | Ostrava | Czech Republic |
| Genetika Plzeň | Plzeň | Czech Republic |
| Centrum Lékařské Genetiky s.r.o., Laboratoř molekulární genetiky | České Budějovice | Czech Republic |
| Laboratoř molekulární biologie a genetiky | České Budějovice | Czech Republic |
| LFG spol. s.r.o., MephaCentrum | Ostrava | Czech Republic |
| Laboratoř molekulární diagnostiky, IFCOR-99, s.r.o. | Brno | Czech Republic |
| Laboratoře AGEL a.s. | Nový Jičín | Czech Republic |
| Transfuzní oddělení, Masarykova nemocnice | Ústí nad Labem | Czech Republic |
| Oddělení klinické biochemie, Krajská nemocnice Liberec | Liberec | Czech Republic |
| AeskuLab a.s. | Praha | Czech Republic |
| Odd. molekulární biologie, Zdravotní ústav se sídlem v Ostravě | Ostrava | Czech Republic |
| Institut reprodukční medicíny a genetiky, s.r.o. | Karlovy Vary | Czech Republic |
| Oddělení lékařské genetiky, FN Brno | Brno | Czech Republic |
| Ústav imunologie, FN Olomouc | Olomouc | Czech Republic |
| Cytogenetická laboratoř Brno, s.r.o. | Brno | Czech Republic |
| Laboratoře HLA systému a PCR diagnostiky, FN Hradec Králové | Hradec Králové | Czech Republic |
| GHC Genetics | Praha | Czech Republic |
| Chemila spol. s r.o. | Hodonín | Czech Republic |
| Klinická imunologie a alergologie, VFN Praha | Praha | Czech Republic |
| Spadia LAB | Nový Jičín | Czech Republic |
| GENLABS s.r.o. | České Budějovice | Czech Republic |
| HLA laboratoř, FN Plzeň | Plzeň | Czech Republic |
| Bioptická laboratoř, s.r.o. | Plzeň | Czech Republic |
| Gennet, s.r.o. | Praha | Czech Republic |
| Elisabeth Pharmacon | Brno | Czech Republic |
| Centrum imunologie a mikrobiologie | Ústí nad Labem | Czech Republic |
| HLA laboratoř, FN Brno | Brno | Czech Republic |
| Oddělení lékařské genetiky, Thomayerova nemocnice | Praha | Czech Republic |
| Laboratoř molekulární genetiky, nemocnice Jihlava | Jihlava | Czech Republic |
| GENvia, s.r.o. | Praha | Czech Republic |
| Genomac výzkumný ústav, s.r.o. | Praha | Czech Republic |
| Laboratoře lékařské genetiky, s.r.o. | Pardubice | Czech Republic |
| Laboratoř molekulární diagnostiky, Nemocnice na Homolce | Praha | Czech Republic |
| Dynex Technologies, s.r.o. | Buštěhrad | Czech Republic |
| Krevní centrum s.r.o. | Frýdek Místek | Czech Republic |
| Synlab czech, s.r.o. | Praha | Czech Republic |
| Fertimed, s.r.o. | Olomouc | Czech Republic |
|  |  |  |

| Oddelenie lekárskej genetiky, FN Trenčín | Trenčín | Slovakia |
| --- | --- | --- |
| Institut für medizinische und chemische Labordiagnostik | Wels | Austria |
| Institut für Laboratoriumsmedizin | St. Pölten | Austria |
| Klinická biochémia, s.r.o. | Žilina | Slovakia |
| Pracownia Typowania Tkankowego Uniwersytecki Szpital Dzieciecy | Krakow | Poland |
| Institut für Laboratordiagnostik, Kaiser-Franz-Josef-Spital | Vienna | Austria |
| Laboratory of Tissue Immunology, Hirszfeld Institute of Immunology and Experimental Therapy, Polish Academy of Sciences | Wroclaw | Poland |
| Department for Tissue Typing and Immunogenetics, Transfusion Center, University Clinical Hospital Mostar | Mostar | Bosnia and Herzegovina |
| Sit Mestre - Laboratorio HLA | Zelarino Mestre | Italy |
| Laboratory of Immunogenetics, Department of Hamatology, Transplantation and Internal Medicine, University clinical Center of the Medical University of Warsaw | Warsaw | Poland |
| Laboratory of Molecular and HLA Diagnostics, University Hospital Centre Osijek | Osijek | Croatia |
|  |  |  |
